# Behavioral Alterations in Mice Carrying Homozygous *HDAC4*^*A778T*^ Missense Mutation Associated With Eating Disorder

**DOI:** 10.3389/fnins.2020.00139

**Published:** 2020-02-21

**Authors:** Kevin C. Davis, Kenji Saito, Samuel R. Rodeghiero, Brandon A. Toth, Michael Lutter, Huxing Cui

**Affiliations:** ^1^Department of Neuroscience and Pharmacology, University of Iowa Carver College of Medicine, Iowa City, IA, United States; ^2^F.O.E. Diabetes Research Center, University of Iowa Carver College of Medicine, Iowa City, IA, United States; ^3^Obesity Research and Educational Initiative, University of Iowa Carver College of Medicine, Iowa City, IA, United States; ^4^Iowa Neuroscience Institute, University of Iowa Carver College of Medicine, Iowa City, IA, United States; ^5^Eating Recovery Center of San Antonio, San Antonio, TX, United States

**Keywords:** eating disorders, histone deacetylase 4, mouse behaviors, social subordination, body weight, food intake, genetic mutation, humanized mouse model

## Abstract

Eating disorders (EDs) are serious mental illnesses thought to arise from the complex gene-environment interactions. DNA methylation patterns in histone deacetylase 4 (*HDAC4*) locus have been associated with EDs and we have previously identified a missense mutation in the *HDAC4* gene (*HDAC4*^*A786T*^) that increases the risk of developing an ED. In order to evaluate the biological consequences of this variant and establish a useful mouse model of EDs, here we performed behavioral characterization of mice homozygous for *Hdac4*^*A778T*^ (corresponding to human *HDAC4*^*A786T*^) that were further backcrossed onto C57BL/6 background. When fed high-fat diet, male, but not female, homozygous mice showed a trend toward decreased weight gain compared to their wild-type littermates. Behaviorally, male, but not female, homozygous mice spent less time in eating and exhibited reduced motivation to work for palatable food and light phase-specific decrease in locomotor activity. Additionally, homozygous *Hdac4*^*A778T*^ female, but not male, mice display social subordination when subjected to a tube dominance test. Collectively, these results reveal a complex sex- and circadian-dependent role of ED-associated *Hdac4*^*A778T*^ mutation in affecting mouse behaviors. Homozygous *Hdac4*^*A778T*^ mice could therefore be a useful animal model to gain insight into the neurobiological basis of EDs.

## Introduction

Eating disorders (EDs), such as anorexia nervosa (AN) and bulimia nervosa (BN), are serious mental illnesses thought to develop as a result of complex gene-environment interaction (Hinney et al., 2010; Carmichael and Lockhart, 2012; Lutter et al., 2016; Baker et al., 2017). In spite of EDs having the highest mortality rate of all mental disorders, available treatments are extremely limited, mainly due to the poor understanding of the underlying neurobiology of EDs and lack of useful animal models that can be used to develop novel treatment strategies (Lutter et al., 2016; Lutter, 2017). Among the proposed factors contributing to EDs, genetics plays a major role in the development of an ED, as family and twin studies indicate that the risk of developing an ED is 60–80% heritable (Lilenfeld et al., 1998; Strober et al., 2000; Karwautz et al., 2002; Baker et al., 2017).

Recent advancement in large scale genetic approaches provide some clues in understanding the genetic basis of EDs (Wang et al., 2011; Boraska et al., 2014; Duncan et al., 2017; Huckins et al., 2018; Watson et al., 2019), but replicable and definitive genetic links to EDs have not yet been established. By combining genetic linkage analysis and whole exome sequencing in two independent families in which multiple members are affected by EDs, we have previously identified two missense mutations in the estrogen-related receptor alpha (*ESRRA*^*R188Q*^) and histone deacetylase 4 (*HDAC4*^*A786T*^) genes, respectively (Cui et al., 2013), that increase the risk of developing an ED. While the family in which *ESRRA*^*R188Q*^ mutation was found has a high rate of AN and comorbidity of obsessive-compulsive disorder (OCD), another family in which *HDAC4*^*A786T*^ mutation was found has higher rates of BN but significantly lower rates of comorbid OCD. Multiple independent studies have shown that many CpG sites located in genomic region around 5′ upstream of *HDAC4* gene are differentially methylated in the peripheral tissues of patients with AN (Booij et al., 2015; Kesselmeier et al., 2018; Subramanian et al., 2018; Sild and Booij, 2019; Steiger et al., 2019), further supporting the role of *HDAC4* in the pathophysiology of EDs. In the followup mouse studies, we found that *Esrra* knockout mice display behavioral deficits relevant to EDs, including reduced food intake, decreased motivation to work for high-fat diet (HFD), and social subordination (Cui et al., 2015). Furthermore, mice heterozygous for *Hdac4*^*A778T*^ [corresponding to the human *HDAC4*^*A786T*^ mutation which we previously reported to be a gain-of-function mutation in the catalytic domain of HDAC4 to increase gene suppression in cultured cells (Cui et al., 2013)] on a mixed C57BL/6 and 129/Sv genetic background display altered feeding behaviors depending on housing condition (Lutter et al., 2017). In order to definitively evaluate the biological consequence of *Hdac4*^*A778T*^ mutation, here we performed a comprehensive behavioral characterization in homozygous *Hdac4*^*A778T*^ mice that we further backcrossed onto C57BL/6 background till reach over 98% of purity. We report that homozygous *Hdac4*^*A778T*^ mice, which are raised by heterozygous *Hdac4*^*A778T*^ dams on a C57BL/6 background, display behavioral alterations that are distinct from heterozygous *Hdac4*^*A778T*^ mice previously reported. Our results may indicate a complex role for *Hdac4*^*A778T*^ mutation in affecting mouse behaviors in different genetic background and growth environment with sex-dependent manner.

## Materials and Methods

### Animal Usage

All animal procedures were performed in accordance with University of Iowa Institutional Animal Care and Use Committee guidelines. Mice were handled in accordance with the Guideline for the Care and Use of Laboratory Animals as adopted by the United States National Institutes of Health. Specific protocols were approved by the Institutional Animal Care and Use Committee. Mice were housed in the University of Iowa vivarium in a temperature-controlled environment (lights on: 06:00–18:00) with *ad libitum* access to water and regular chow (7913 NIH-31 modified open formula mouse sterilized diet, Harlan-Teklad) or 60% HFD (Research Diet, #D12492) as noted.

### Generation of *HDAC4*^*A778T*^ Mice

**FIGURE 1:**
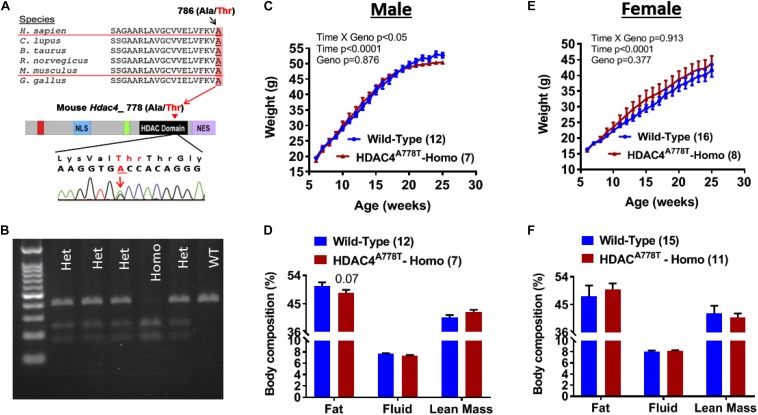
The effects of *Hdac4*^*A778T*^ mutation on body weight and body composition. **(A)** Schematic drawing of conserved human HDAC4^*A786T*^ mutation across the species and corresponding mouse *Hdac4*^*A778T*^ mutation in the catalytic domain of HDAC4. **(B)** Representative genotyping image of *Hdac4*^*A778T*^ mutation by agarose gel electrophoresis of BStEII-digested PCR product. **(C–F)** Male mice body weight **(C)** and body composition, and female mice body weight **(E)** and body composition **(F)** on HFD. Data are presented as mean ± SEM.

*Hdac4*^*A778T*^ point mutant knock-in mouse was generated by introducing *Hdac4*^*A778T*^ mutation that correspond to human HDAC4^*A786T*^ mutation (Figure 1A) by CRISPR technology as previously reported (Lutter et al., 2017). Heterozygous mice with mixed C57BL/6 and 129/Sv genetic background were further backcrossed to C57BL/6 female mice until reach over 98% of C57BL/6 background. Male and female heterozygous *Hdac4*^*A778T*^ mutant mice were bred to generate wild-type (WT) and homozygous *Hdac4*^*A778T*^ mice for experimental use. Mice were weaned at 3 weeks and genotyped by PCR amplification of tail genomic DNA encompassing HDAC4^*A778T*^ mutation (primer pairs used: 5′-TCAGCCACTGGGAAGGTTAG-3′ and 5′-AGCCCAGGTCTGTTAGAGCA-3′) followed by restriction enzyme digestion (*Bst*EII-HF, New England Biolab) at 37°C for 2 h. Genotypes were determined by running BStEII-digested PCR product in agarose gel as shown Figure 1B.

### Body Weight Gain and Body Composition on High-Fat Diet (HFD)

An independent cohort of mice was produced for metabolic phenotyping on HFD. Mice were weaned and genotyped by tail snip and PCR at 3 weeks of age. Group-housed mice were fed 60% HFD (Research Diet, #D12492) for 20 weeks beginning at 6 weeks of age and body weight was monitored weekly. At the end of the study, body composition for some of mice was determined by the University of Iowa Metabolic Phenotyping Core using a Bruker Minispec LF50 (Bruker, Billerica, MA, United States).

### Behavioral Studies

Two different cohorts of seven to 12-week-old mice kept on a normal chow diet were used for behavioral studies. The first large cohort of male and female mice were sequentially (in order of least to most stressful) subjected to rotarod, elevated plus maze test, open field test, light/dark box test, LABORAS test, social interaction test, social dominance test, sucrose preference test, tail suspension test, forced swim test, and operant responding test. A second independent cohort of male and female mice were produced and subjected to binge-like eating behavioral paradigm.

#### Rotarod

Mice were placed on a rotarod apparatus (IITC Life Science, Woodland Hills, CA, United States) and the rotational speed was ramped up from an initial 4 rpm to 40 rpm in 4 min. Mice were recorded in sets of five mice at a time. The time for each mouse to fall off the rotarod was measured for three trials consecutively before moving on to the next set of mice. Mice were then placed back into their original home cages for ∼24 h. Data for each trial was averaged to generate an average daily score for each mouse. The test was repeated over the next 2 days for a total of 3 days of testing.

#### Elevated Plus Maze

Mice were subjected to the elevated plus maze (EPM) test to test for anxiety-like behavior as previously reported (Cui et al., 2015; Lutter et al., 2017). Mice were brought to the testing room approximately 1 h prior to testing to allow time for mice to acclimate to the testing environment. Mice were placed in the center of the apparatus and allowed to explore for 5 min during which the mice were tracked using ANY-Maze Behavioral tracking software (Stoelting, Wood Dale, IL, United States). The time spent in the center, the open or closed arms were measured and analyzed.

#### Open Field Test

Mice were subjected to the open field test to evaluate for novel environment exploration and anxiety-related behavior as previously reported (Cui et al., 2015; Lutter et al., 2017). Mice were placed in the testing room approximately 1 h prior to testing to allow time for the mice to acclimate to the testing environment. Each mouse was placed in the center of an open box (42 cm × 42 cm) and allowed to explore for 5 min. Subjects were recorded and time spent in the center of the box (20 cm × 20 cm^2^) and time spent in the edges of the field was recorded. Data was collected using ANY-Maze behavioral tracking software (Stoelting, Wood Dale, IL, United States).

#### Light-Dark Box Test

The Light-Dark box test was performed under dimmed light conditions as reported previously (Cui et al., 2015; Lutter et al., 2017). During 10 min of testing, the time spent on the light side of the box and distance traveled was calculated using ANY-maze behavioral tracking software (Stoelting).

#### Laboratory Animal Behavior Observation Registration and Analysis System (LABORAS)

Mice were placed in an automated LABORAS system (Metris, Netherlands), to quantify the locomotion and the time spent on eating, drinking, climbing, grooming, and rearing in a home cage-like environment as previously reported (Cui et al., 2015; Lutter et al., 2017). Mice were placed in the cage shortly after the beginning of the light cycle and the system was left to collect data for up to 24 h. To allow the system to accurately track and measure the animal’s behavior, mice were singly-housed in these cages for the duration of the test. The data were analyzed and compared separately for light and dark cycles.

#### Three-Chamber Sociability and Social Novelty Test

The social interaction test was performed as previously described (Cui et al., 2015; Lutter et al., 2017). Briefly, two novel female and male mice were selected as novel mice and allowed to acclimate for 5 min each in a small cage within a three-chambered apparatus. Each test mouse was then given 5 min to explore the three chambers. The test mouse was then moved to the middle chamber and trapped by closing the doors to the left and right chambers. The novel mouse was then placed in a side chamber, the doors were removed and the test mouse was allowed to explore the apparatus for 10 min. The test mouse was then moved back and trapped in the middle chamber, and the second novel mouse was then placed in the chamber opposite the initial novel mouse. The doors were removed and the mouse was again allowed another 10 min to explore the apparatus. The side that the novel mouse is placed on was alternated in between each test mouse. Behavioral activity was recorded and measured using ANY-Maze behavioral tracking software (Stoelting).

#### Social Dominance Test

Tube dominance test was performed as previously reported (Cui et al., 2015; Lutter et al., 2017). Each mouse under went 3 days of training prior to the test day. During the first training day, each mouse was guided into a three foot-long tube with an inner-diameter of 3/8-inches. A dark enclosure (the goal box) was fixed at the other end of the tube and the mouse was given 180 s to reach the goal box. This process was repeated for each mouse on the first day of training. On the subsequent training days, each mouse performed the same task twice as done on first day training, but each subject was only given 30 s to reach the goal box. During training, the subject was forced to the goal box after the time expired. On test day, wild-type and mutant mice were set up in pairs in a round-robin tournament ensuring that mice in each pair had never previously interacted. A partition was placed in the middle of the tubing and each mouse of each pair was placed at opposite ends of the tubing. Once mice reached the middle of the tube the partition was removed. The inner-diameter of the tube was such that mice were unable to turn around. The first mouse to retreat from the tubing was categorized as the loser, and the other as the winner.

#### Sucrose Preference Test

The sucrose preference test can assay for anhedonia in mice, which can be used as an associative measurement of depression. Mice were singly-housed and given free access to either a 2% sucrose solution or water. The solutions were placed in 50 mL conical tubes that were fitted with an animal drinking nozzle in a rubber stopper. Amounts of solution taken were measured over 4 days, during which the location of the bottles was switched every day to avoid place preference. Amounts were taken by change in weight. The data were only analyzed for the last 2 days were used for the data analysis. Sucrose preference was calculated by the ratio of amount of sucrose taken over total solution consumed and compared between the groups.

#### Tail Suspension Test

Tail suspension behavioral despair test was performed to evaluate depression-like symptoms as previously reported (Castagne et al., 2010). Each mouse had its tail taped to a hook and was left hanging for 6 min. Video of the subject’s movements and behavior was recorded using ANY-maze behavioral tracking software. The videos were then manually scored for time that each mouse spent immobile and compared between the groups.

#### Forced Swim Test

Test was performed as described previously (Castagne et al., 2010; Cui et al., 2015; Lutter et al., 2017). ANY-Maze behavioral tracking settings were identical to the previous study.

#### Operant Responding Test

Mice were tested for motivation for high-fat diet through operant responding as previously described (Cui and Lutter, 2013; Cui et al., 2015; Lutter et al., 2017). Briefly, mice were trained to press a lever for a high-fat diet pellet reward. Mice were trained to pass one fixed-ratio (FR) 1 program, two FR 3 programs, and three FR 5 programs prior to testing. Mice were fasted for ∼20 h prior to each training session. Testing consisted of 6 days. During the first three test days, mice continued to be fasted for ∼20 h and were placed on progressive ratio schedules. Mice were given ad lib feeding on days 4–6. The operant conditioning apparatus was cleaned with Windex after uses.

#### Binge Eating Test

Finally, binge eating behavior was assessed in mice. Another cohort of mice were singly-housed and given free access to both a high-sugar high-fat diet (Research Diets, New Brunswick, NJ, United States), and regular chow. The test was performed in a similar manner to methods previously performed (Czyzyk et al., 2010). Diets dispensed in two separate food hoppers on either the left or right side of the cage top. The side that the food was placed on was randomized. The testing consisted of seven cycles lasting a week at a time. Food intake data was collected each week 2.5 and 24 h after reintroducing the mice to HFD each week. After each 24 h recording, HFD was removed but mice were still kept on regular chow during the remainder of the week, until the cycle would start again and mice were reintroduced.

### Statistical Analyses

Statistical analyses were performed using GraphPad Prism (GraphPad Software, La Jolla, CA, United States) software. Comparisons between groups were made by Student’s *t*-test (anxiety-like behavior, depression-like behaviors, and social dominance test), multiple *t*-test (body composition and LABORAS), one-way (three-chamber sociability test) or repeated measure two-way ANOVA (Rotarod, body weight, operant responding test, and binge-like eating with genotype and time as independent variables) with Sidak *post hoc* analysis as needed. *P* < 0.05 was considered statistically significant. Data are presented as mean ± SEM.

## Results

### The Effects of *Hdac4*^*A778T*^ Mutation on Body Weight and Body Composition

Male and female mice heterozygous for *Hdac4*^*A778T*^ mutation (>98% of C57BL/6 genetic background) were bred together to generate experimental cohorts of mice. Pups were then weaned and genotyped at 3 weeks of age (Figure 1B). At 6 weeks of age, group-housed mice were given access to 60% high-fat diet (HFD) and body weights were recorded until 26 weeks of age. Group-housed homozygous male mice showed slight but significant reduction in body weight gain compared to their WT littermates (Genotype × Time interaction with repeated measure two-way ANOVA, *F*_19_,_323_ = 1.853, *p* = 0.017, Figure 1C) without significant change of body composition (Figure 1D), whereas female homozygous mice did not show statistical significance (Genotype × Time interaction with repeated measure two-way ANOVA, *F*_19_,_399_ = 0.591, *p* = 0.913, Figure 1E). No significant change in body composition was observed for female mice (Figure 1F).

### The Effects of *Hdac4*^*A778T*^ Mutation on Motor Coordination and Anxiety- and Depression-Like Behaviors

**FIGURE 2:**
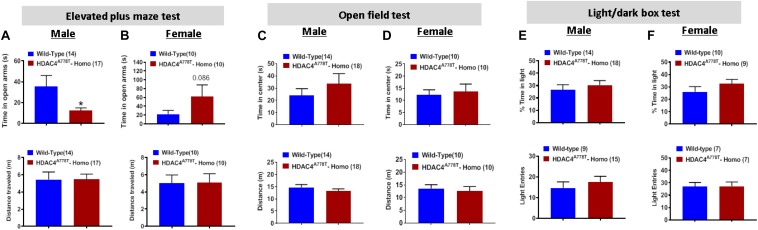
The effects of *Hdac4*^*A778T*^ mutation on anxiety-like behaviors. **(A,B)** The time spent in open arms and total distance traveled during EPM test for both male **(A)** and female **(B)** mice. **(C,D)** The time spent in center zone and total distance traveled during OF test for both male **(C)** and female **(D)** mice. **(E,F)** The% of time spent in light box and the number of entries to light box during L/D test for both male **(E)** and female **(F)** mice (*p < 0.05). Data are presented as mean ± SEM.

To first determine whether mice homozygous for *Hdac4*^*A778T*^ mutation have impaired motor coordination, we performed the rotarod test and found no difference in motor performance between WT and homozygous mice for both sexes (Supplementary Figures S1A,B). Mice were then subjected to three different behavioral paradigms frequently used to determine levels of anxiety in mice: elevated plus maze test (EPM), open field test (OF), and light/dark box test (LDB). For EPM, homozygous male mice spend less time on open arms compared to their WT littermates (by Student *t*-test, *t*_(29)_ = 2.27, *p* = 0.0154, Figure 2A), while homozygous female mice tend to spend more time on open arms though it did not reach statistical significance (Figure 1B). Distance traveled during 5 min of EPM test was comparable between the groups for both male and female mice (Figures 2A,B). For OF test, the time spent in center zone and the distance traveled during the test was comparable between the groups for both sexes (Figures 2C,D). Likewise, no difference was observed for the time spent in light box or the number of entries to light box between the groups for both sexes (Figures 2E,F).

**FIGURE 3:**
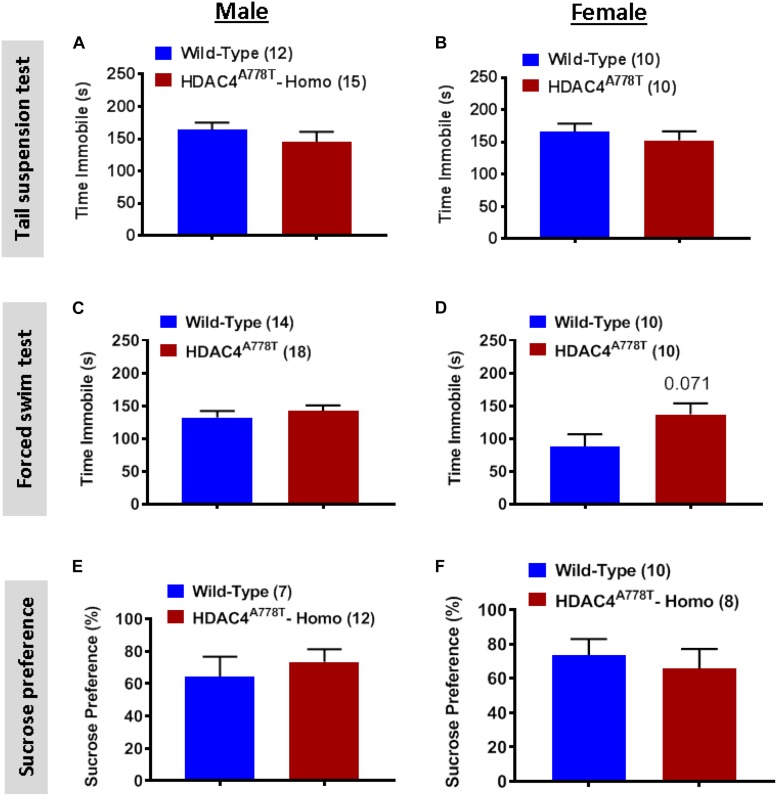
The effects of homozygous *Hdac4*^*A778T*^ mutation on depression-like behaviors. **(A,B)** Immobile time during tail suspension test for both male **(A)** and female **(B)** mice. **(C,D)** Immobile time during tail suspension test for both male **(C)** and female **(D)** mice. **(E,F)** % of sucrose consumed during sucrose preference test for both male **(E)** and female **(F)** mice. Data are presented as mean ± SEM.

We also subjected mice to three well-established behavioral paradigms used to evaluate the levels of depression-like symptoms: tail suspension test (TST), forced swim test (FST), and sucrose preference test (SPT). For all three tests, we did not observe any significant differences between the groups for both sexes (Figures 3A–F), except female homozygous mice showed a non-statistically significant increase in immobile time during FST compared to their WT littermates (Figure 3D).

### The Effects of *Hdac4*^*A778T*^ Mutation on Behaviors in Home Cage-Like Environment (LABORAS)

**FIGURE 4:**
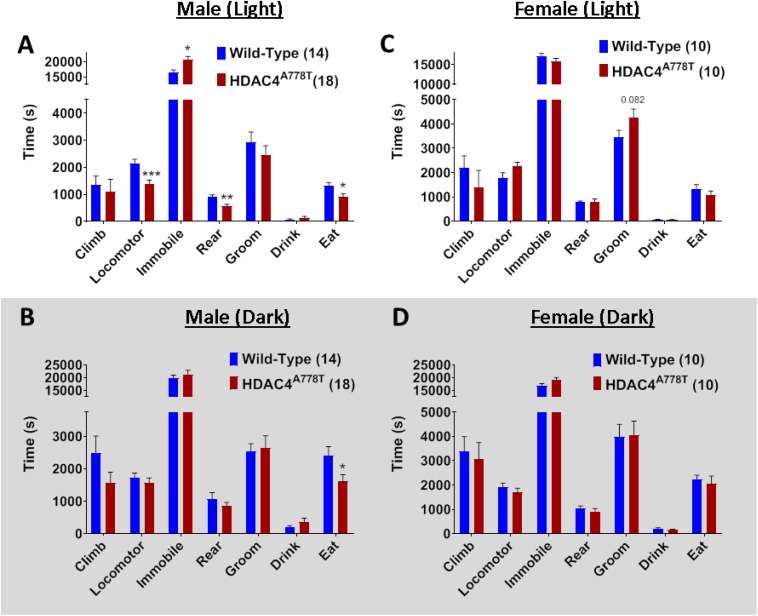
The effects of homozygous *Hdac4*^*A778T*^ mutation on general behaviors in home cage-like environment. **(A,B)** Vibration-based LABORAS behavioral quantification for male mice during light **(A)** and dark **(B)** cycles. **(C,D)** Vibration-based LABORAS behavioral quantification for female mice during light **(C)** and dark DB) cycles (*p < 0.05, **p < 0.01, ***p < 0.001). Data are presented as mean ± SEM.

Laboratory Animal Behavior Observation Registration and Analysis System has been used to analyze mouse behaviors in home cage-like environment for extended period of time as we have previously reported (Cui et al., 2015; Lutter et al., 2017). Singly housed male and female mice were allowed to spend overnight (∼24 h) in LABORAS cage and the data were collected and analyzed separately for light and dark phase. Male homozygous mice overall spend significantly less time in eating (by multiple *t*-test, *t*_(30)_ = 2.19, *p* = 0.036, Figure 4A), but not drinking, during both light and dark phases, while they display light cycle-specific reduction in locomotor activity (by multiple *t*-test, *t*_(30)_ = 3.898, *p* = 0.0005, Figure 4A) and rearing behavior (by multiple *t*-test, *t*_(30)_ = 3.083, *p* = 0.004, Figure 4A). No differences were observed in female homozygous mice, except a trend toward increased grooming in light cycle (Figures 4C,D).

### The Effects of *Hdac4*^*A778T*^ Mutation on Social Behaviors

**FIGURE 5:**
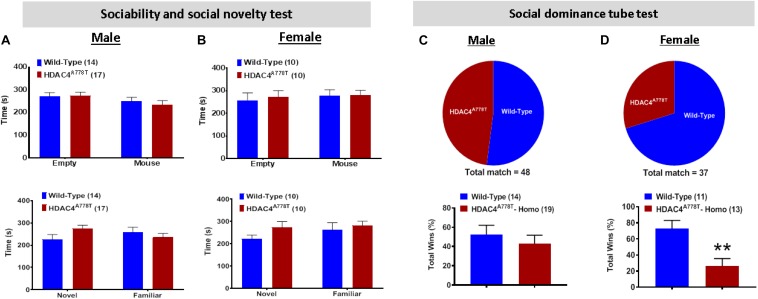
The effects of homozygous *Hdac4*^*A778T*^ mutation on socia behaviors. **(A,B)** Time either spent in empty box or interacted with familiar or novel mice during Immobile time during three-chamber sociability and social novelty test for both male **(A)** and female **(B)** mice. **(C,D)** % of total win during a round-robin tournament of tube dominance test for both male **(C)** and female **(D)** mice (**p < 0.01). Data are presented as mean ± SEM.

Impaired social behaviors have been repeatedly reported in patients with EDs. To determine whether *Hdac4*^*A778T*^ mutation has an impact on social functions, we subjected the mice to three-chamber sociability and social novelty test as well as social dominance tube test as previously reported (Cui et al., 2015; Lutter et al., 2017). Both male and female WT and mutant mice exhibited no significant difference for both social interaction and social novelty test during 3-chamber sociability test (Figures 5A,B). However, for social dominance tube test, female homozygous mice exhibited a fewer number of wins or a greater number of retreats from the tube (by Student *t*-test, *t*_(22)_ = 3.318, *p* = 0.003, Figure 5C), which was not observed in males (Figure 5D). This result indicates social subordination in female homozygous mice compared to their WT littermates.

### The Effects of *Hdac4*^*A778T*^ Mutation on Food Reward Behavior

**FIGURE 6:**
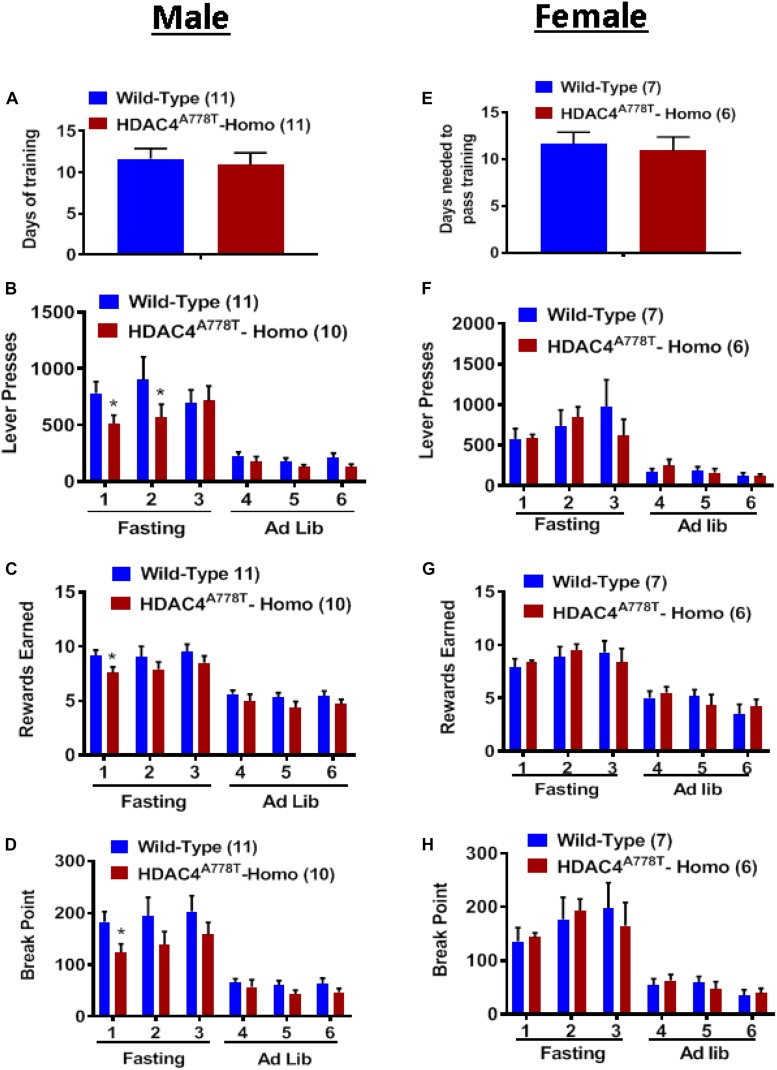
The effect of homozygous *Hdac4*^*A778T*^ mutation on food motivation. **(A)** Days needed to pass all required FR training in male mice. **(B–D)** Total numbers of lever press **(B)**, total numbers of reward earned **(C)**, and calculated break point **(D)** during operant responding test with PR schedule for male mice. **(E)** Days needed to pass all required FR training in female mice. **(F–H)** Total numbers of lever press **(F)**, total numbers of reward earned **(G)**, and calculated break point HD) during operant responding test with PR schedule for female mice (*p < 0.05). Data are presented as mean ± SEM.

Food-associated reward dysfunction is one of core features of patients with EDs (Fladung et al., 2010; Frank et al., 2016; Foldi et al., 2017). To determine whether mice carrying homozygous *Hdac4*^*A778T*^ mutation have impaired food-associated reward behavior, we performed effortful operant responding test using a progressive ratio schedule; a well-established behavioral paradigm often used to determine the desire and the motivation for an animal to obtain palatable food. As described in the method section, the mice first had to learn and pass all required training (FR1 × 1, FR3 × 2, and FR5 × 3) before they enter PR schedule for motivation. On average, both male and female homozygous mice took a similar amount of time as WT to pass all required training, indicating that procedural learning ability in homozygous mice is intact (Figures 6A,E). Subsequent measurement for food motivation with PR schedule reveal that male, but not female, homozygous mice tend to less motivated to press lever (by multiple *t*-test, *t*_(119)_ = 2.027, *p* = 0.044, Figure 6B; *t*_(19)_ = 2.28, *p* = 0.034, Figure 6D) to obtain high-fat pellets (by multiple *t*-test, *t*_(19)_ = 2.188, *p* = 0.041, Figure 6C) after overnight fasting. No significant differences were observed when mice were fed ad lib (Figures 6B–D, F–H).

### The Effects of *Hdac4*^*A778T*^ Mutation on Binge-Like Eating

**FIGURE 7:**
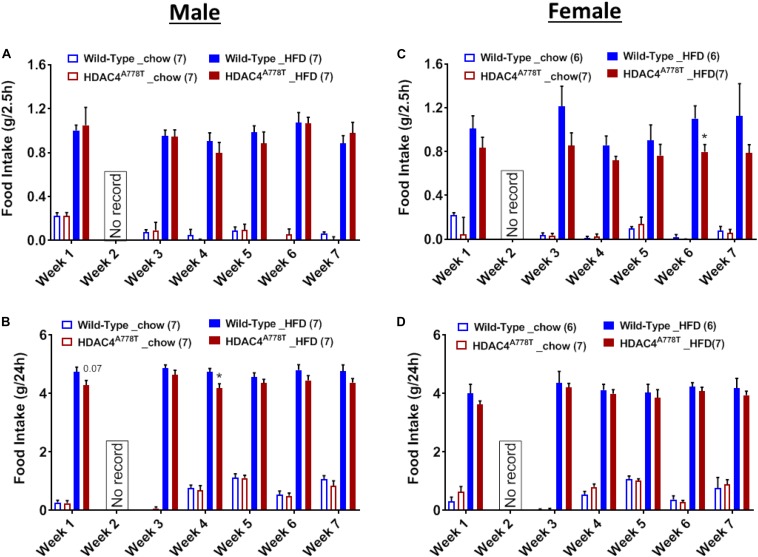
The effects of homozygous *Hdac4*^*A778T*^ mutation on binge-like eating. **(A,B)** 2.5-hour **(A)** and 24-hour **(B)** food intake during 7 weeks of binge-like eating test for male mice. **(C,D)** 2.5-hour **(C)** and 24-hour **(D)** food intake during 7 weeks of binge-like eating test for male mice (*p < 0.05). Data are presented as mean ± SEM.

Binge-like eating behavior assessed by the intake of HFD during first 2.5 h of intermittent access to HFD revealed that female, but not male, homozygous mice tend to have reduced binge-like eating behavior compared to their WT littermates (Figures 7A,C). However, 24-hour intake of HFD during weekly intermittent access reveal that male, but not female, homozygous mice tend to consume less amount of HFD compared to their WT littermates (Figures 7B,D).

The major metabolic and behavioral differences observed in homozygous mice of current study and heterozygous mice of our previous report are summarized in Table 1.

**TABLE 1:** Summary of phenotypes observed in homozygous and heterozygous mice.

		Homozygous (C57/BL6 background)		Heterozygous (Mixed background)	
		Male-group	Female-group	Male-group	Female-group
Body weight	Chow	ND	ND	–	–
	HFD	↓	–	–	↑
Anxiety-like behavior	Light/dark box (Time in light box)	–	–	–	–
	Open field (Time in center)	–	–	ND	ND
	Elevated plus maze (Time on open arms)	↓	–	–	↓
Depression-like behavior	Forced swim test (Immobility)	–	–	–	↓
	Tail suspension test	–	–	ND	ND
	Sucrose preference	–	–	ND	ND
Social behaviors	Social interaction	–	–	ND	–
	Social dominance	–	↓	–	–
LABORAS	Locomotion	↓	–	–	–
	Grooming	–	–↑	–	↑
	Eating	↓	–	–	↓
Food reward	Operant responding	↓	–	–	↓
	Binge eating	–	*–*↓	ND	ND

*ND, not determined; -↑, marginal increase; -↓, marginal decrease.*

## Discussion

Eating disorders are severe but inadequately treated mental illnesses, in part due to our limited understanding of underlying neurobiology of the illness as well as lack of reliable animal models that can be used for rigorous testing and development of treatment options (Lutter, 2017). As an attempt to establish a more reliable animal model of EDs, in the present study we performed extensive behavioral characterization of homozygous mice for the *Hdac4*^*A778T*^ missense mutation, which we previously found to be associated with the risk of developing an ED (Cui et al., 2013). We observed distinct behavioral phenotypes compared to previously reported phenotypes for heterozygous *Hdac4*^*A778T*^ mice (Lutter et al., 2017). One notable difference is the gender for which the phenotypes were observed. In contrast to the majority of phenotypes observed in female heterozygous *Hdac4*^*A778T*^ mice, in the present study behavioral abnormalities were mainly observed in male homozygous mice (Table 1). While it is puzzling how differences in genotype (homo vs. het) and genetic background (C57BL/6 vs. mixed) affect behavioral phenotypes in different sexes, links between estrogenic signaling and HDAC4 have been well documented. For instance, it has been shown that HDAC4 can be recruited by the N-terminal region of estrogen receptor alpha (ERα) to the promoter of endogenous estrogen responsive genes and suppresses transcriptional activity of ERα in a cell type-dependent manner (Leong et al., 2005); HDAC2 and HDAC4 have been reported to involve in epigenetic histone modifications of ERα, which affect the developmental process of brain masculinization (Matsuda et al., 2011); and more recently, it was shown that *HDAC4* is associated with female post-traumatic stress disorder (PTSD) likely through a direct effect of estrogen on *HDAC4* expression during traumatic stress (Maddox et al., 2018). Future studies focusing on the bidirectional association between *HDAC4* and estrogenic signaling may advance our understanding of neurobiological basis of EDs.

Another factor that may have an impact on the different behavioral phenotypes observed is the “environment” in which all experimental cohorts of mice were produced. It is noteworthy that, compared to WT dams from which all experimental cohort of WT and heterozygous *Hdac4*^*A778T*^ mutant mice were produced in our previous report (Lutter et al., 2017), all experimental cohorts of WT and homozygous *Hdac4*^*A778T*^ mice in the present study were produced and raised by *Hdac4*^*A778T*^ heterozygous dams, which showed notable social deficits in the previous study (Lutter et al., 2017). Therefore, the maternal environments in which the experimental mice grew up, including *in utero* and pre-weanling postnatal growth, are different between the two studies. While it is known that gene-environmental interactions and epigenetics play an important role for the development of an ED (Campbell et al., 2011; Strober et al., 2014; Steiger and Thaler, 2016; Thaler and Steiger, 2017), it is impossible to distinguish whether the behavioral alterations observed in the present study are due to either genetics or environmentally mediated epigenetic regulations (or could be a combination of both). Future studies with a cross-fostering design may help to evaluate the effect of pre-weanling postnatal growth environment on behavioral manifestations associated with *Hdac4*^*A778T*^ mutation.

One interesting behavioral phenotype that we observed for homozygous *Hdac4*^*A778T*^ mutant mice, but not for heterozygous mice, is social subordination. Compared to WT littermates, most female, but not male, homozygous mice are submissive in a tube dominance test, which is consistent with absolute social subordination we previously observed in female *Esrra-null* mice – another mutation that was also found to be associated with EDs (Cui et al., 2013). These observations, along with clinical findings showing that many patients with EDs have social deficits (Troop et al., 2003, 2014; Connan et al., 2007; Troop and Baker, 2008; Veronese et al., 2010), suggest that the trait of social subordination may represent a useful endophenotype of EDs to guide and advance future preclinical studies. Investigating neural circuits and molecular pathways that impact social subordination might therefore open up new avenues for developing a targeted treatment option for EDs.

In summary, we report that homozygous *Hdac4*^*A778T*^ mice on a nearly isogenic C57BL/6 background exhibit certain metabolic and behavioral phenotypes relevant to EDs in a sex-dependent manner, including altered feeding and body weight, reduced motivation for palatable food and social subordination, which may be influenced by complicated environmental factors. Since *HDAC4* has been implicated in many different brain functions, including learning and memory (Kim et al., 2012; Sando et al., 2012; Fitzsimons et al., 2013; Makinistoglu and Karsenty, 2015; Wu et al., 2016), neurodegeneration (Majdzadeh et al., 2008; Li et al., 2012; Mielcarek et al., 2013, 2015; Whitehouse et al., 2015; Wu et al., 2017; Federspiel et al., 2019), drug addiction (Griffin et al., 2017; Penrod et al., 2018; Gonzalez et al., 2019), PTSD (Maddox et al., 2018; Saha et al., 2019), and major depressive disorder (Hobara et al., 2010; Sarkar et al., 2014; Higuchi et al., 2016), future mechanistic investigations in homozygous *Hdac4*^*A778T*^ mice at molecular, cellular and circuit levels will not only provide novel insight into the neurobiological basis of an ED, but also advance our knowledge about the underlying mechanisms of other neuropsychiatric disorders.

## Data Availability Statement

All datasets generated for this study are included in the article/Supplementary Material.

## Ethics Statement

The animal study was reviewed and approved by University of Iowa Animal Care and Use Committee.

## Author Contributions

KD, ML, and HC conceived and designed the experiments and wrote the manuscript. KD, KS, SR, and BT performed the experiments. KD, KS, and HC analyzed the data. HC contributed to reagents, materials, and analysis tools.

## Conflict of Interest

The authors declare that the research was conducted in the absence of any commercial or financial relationships that could be construed as a potential conflict of interest.
