# Supplementary material for: Behavioral Alterations in Mice Carrying Homozygous HDAC4A778T Missense Mutation Associated With Eating Disorder
Source: Front Neurosci. 2020 Feb 21;14:139. doi: 10.3389/fnins.2020.00139 (PMC7046559; doi:10.3389/fnins.2020.00139)
Supplement: Supplementary file 1 [file Image_1.pdf]

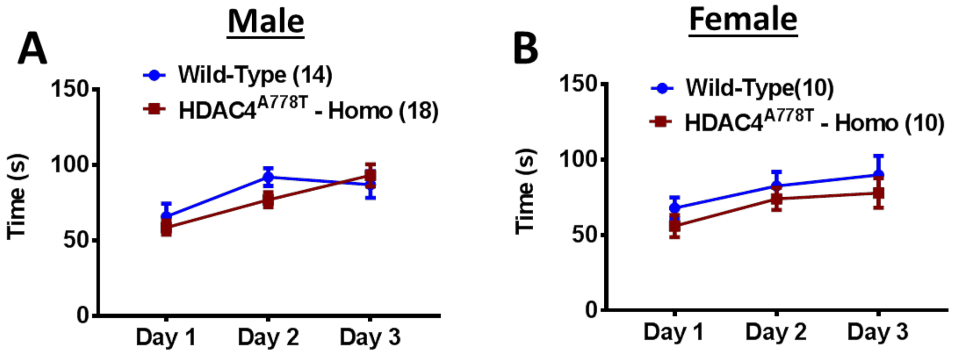

Supplemental Figure 1. The effect of *Hdac4^{A778T}* mutation on motor coordination. Both male (A) and female (B) mice are subjected to rotarod test for 3 consecutive days to evaluate motor coordination. Data are presented as mean ± SEM.
